# Percutaneous drainage of iliopsoas abscess: an effective option in cases not suitable for surgery

**DOI:** 10.1590/S1679-45082018RC4254

**Published:** 2018-09-11

**Authors:** Diego Lima Nava Martins, Francisco de Assis Cavalcante, Priscila Mina Falsarella, Antonio Rahal, Rodrigo Gobbo Garcia

**Affiliations:** 1 Hospital Israelita Albert Einstein, São Paulo, SP, Brazil

**Keywords:** Psoas abscess/diagnostic imaging, Drainage, Ultrasonography, Tomography, x-ray computed, Minimally invasive procedures, Abscesso do psoas/diagnóstico por imagem, Drenagem, Ultrassonografia, Tomografia computadorizada por raios X, Procedimentos cirúrgicos minimamente invasivos

## Abstract

The aim of this study were to describe the technique of percutaneous drainage of iliopsoas abscess, and to discuss the benefits of using this minimally-invasive tool. A single center study with retrospective analysis of patients with psoas abscess confirmed by imaging scans, sent to the interventional medicine center and submitted to computed tomography and ultrasound-guided percutaneous drainage, from November 2013 to August 2016. Seven patients underwent percutaneous drainage of psoas abscess in this period. The mean initial drained volume was 61.4±50.7mL (ranging from 10 to 130mL), and the mean drainage duration was 8.3±2.8 days (ranging from 4 to 12 days). The success rate of the percutaneous procedures was 71.5%, and two patients required re-intervention. Image-guided percutaneous drainage of iliopsoas abscess is a minimally invasive, efficient and safe procedure, and an extremely valuable technique, especially for patients who are not suitable for surgical repair.

## RESUMO

Os objetivos deste estudo foram descrever a técnica de drenagem percutânea de abscesso de iliopsoas e discutir os benefícios do uso desta ferramenta minimamente invasiva. Trata-se de estudo unicêntrico com análise retrospectiva de pacientes com abscesso de psoas confirmado por exames de imagem, encaminhados a um centro de medicina intervencionista e submetidos à drenagem percutânea guiada por tomografia computadorizada e ultrassonografia, de novembro de 2013 a agosto de 2016. Sete pacientes foram submetidos à drenagem percutânea de abscesso de psoas no período avaliado. O volume drenado inicial médio foi 61,4±50,7mL (variação de 10 a 130mL), e a duração média da drenagem foi de 8,3±2,8 dias (variação de 4 a 12 dias). A taxa de sucesso dos procedimentos percutâneos foi 71,5%, e dois pacientes necessitaram de reintervenção. A drenagem percutânea guiada por imagem de abscesso de iliopsoas é um procedimento minimamente invasivo, eficiente e seguro, além de ser uma técnica muito valiosa, especialmente em pacientes que não são candidatos à abordagem cirúrgica.

## INTRODUCTION

Iliopsoas abscess (IPA) consists of a fluid collection within the compartment of the psoas and iliacus muscles.^(^ ^1^ ^)^ In the past, the most frequent etiologies were tuberculous infections of the spine; but with the development of anti-tuberculosis drugs, nontuberculous pyogenic IPA has become the predominant forms.^(^ ^2^ ^)^

The triad of fever, flank pain and limitation of hip movement is observed in only 30% of patients. Most of them present with non-specific general symptoms, like pain, fever, anorexia and weight loss.^(^ ^3^ ^)^

Computed tomography (CT) is useful for diagnosis of iliopsoas abscesses, and contrast-enhanced magnetic resonance (MR) has become the standard modality for diagnosis and follow-up of patients with associated spine diskitis and osteomyelitis, especially in those with epidural and intradural infections.^(^ ^4^ ^)^

Treatment for IPA is controversial, and ranges from immobilization combined with antibiotic or antituberculosis therapy, to surgery, which basically consists of abscess drainage, removal of necrotic tissue and thecal sac decompression, whenever necessary.^(^ ^5^ ^)^

Percutaneous ultrasound- (US) or CT-guided drainage has been described as an effective and low-cost treatment option.^(^ ^4^ ^)^

The objective of this study is to describe the percutaneous drainage of IPA technique and discuss its benefits.

## CASE REPORTS

A single center study with retrospective analysis of patients with psoas abscess, referred to an interventional medicine center and submitted to CT- or US-guided percutaneous drainage, from November 2013 to August 2016. The multidisciplinary team comprising clinicians and surgeons assessed the clinical status of patients and the imaging findings that allowed the percutaneous approach, and indicated the procedures.

Patients followed the preoperative protocol of the hospital (8 hour fasting, RNI below 1.50, and platelet levels greater than 50,000/mL). Decision regarding the type of anesthesia was made considering clinical conditions and patient cooperation.

An initial low-dose CT was performed for access planning, and thin axial CT slices (1.25mm) with multiplanar reconstructions were performed to guide the path of the needle (Chiba 18G/15cm) and its positioning inside the collection. After the proper positioning of the needle and fluid aspiration through needle lumen, drainage was performed as per Seldinger technique, followed by fixation of the drain to the skin, and connection to a collector bag. In all patients, microbiological analysis of the fluid was made. The drains used were Dawson-Mueller (Cook Medical, Bloomington, Ind., United States) or Sump (Bard Medical, Covington, GA, United States).

Seven patients (six males) underwent percutaneous drainage of psoas abscess in the evaluated period. Mean age was 65.1 years (22 to 92 years), and the major symptoms presented at admission were abdominal pain and fever. Patients were clinically evaluated, and then submitted to imaging studies (CT in four cases, US in two cases, and lumbar spine MR in one case).

The average initial drained volume was 61.4±50.7mL (10 to 130mL), and the mean drainage duration was 8.3±2.8 days (4 to 12 days). In two cases, intra-drain alteplase (rt-PA, recombinant tissue-type plasminogen activator) was necessary, for drainage optimization.

Among the seven cases evaluated, six had positive cultures, and the agents isolated were *Pseudomonas aeruginosa* (n=3), *Salmonella spp.* (n=1), *Staphylococcus aureus* (n=1) and *Escherichia coli* (n=1). One patient had negative cultures.

**Figure 1:**
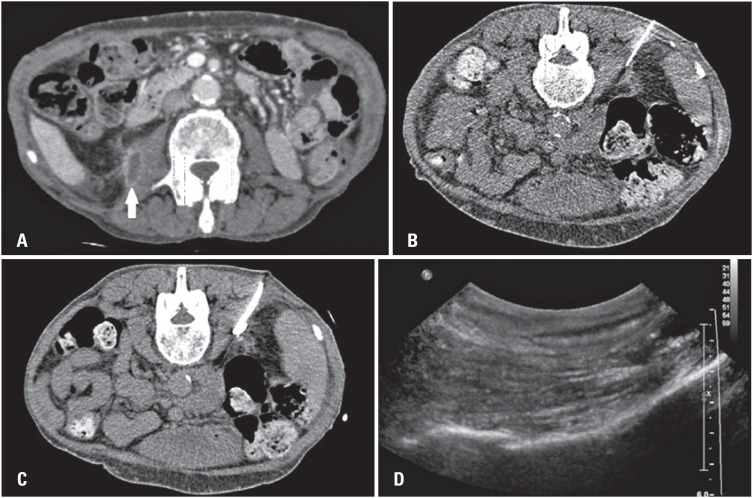
Percutaneous computed tomography-guided drainage using Seldinger technique. (A) Contrast-enhanced abdominal computed tomography showed a small fluid collection inside right psoas (arrow), (B) Final imaging control demonstrating well positioned Dawson-Mueller drain

Figure 1 shows a 76 year-old male patient, with a recent history of bilateral nephrectomy, admitted at emergency department with fever, abdominal and back pain. Contrast-enhanced abdominal CT showed a fluid collection inside right psoas. Percutaneous CT-guided drainage was performed using Seldinger technique, with immediate aspiration of 35mL of purulent fluid. After 9 days, drain was removed due to absence of fluid collections around it. Bacteriological analysis was positive for P. *aeruginosa.*

**Figure 2:**
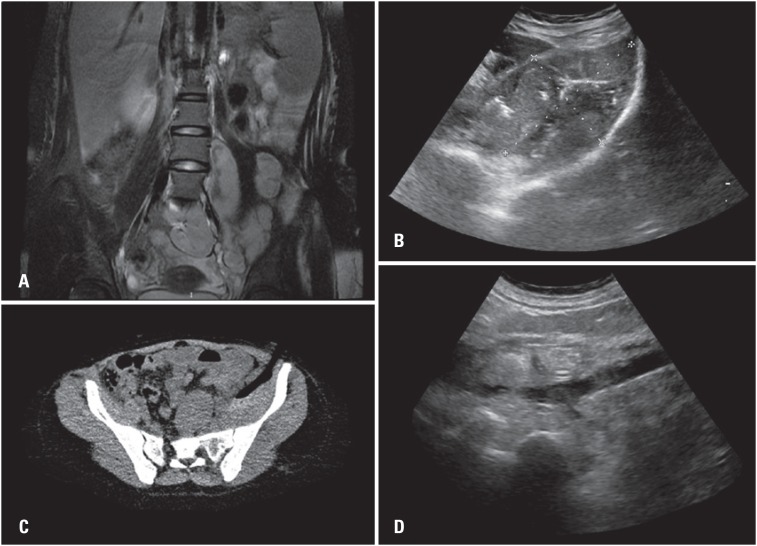
Lumbar spine magnetic resonance imaging, T2-weighted. (A) demonstrates elongated fluid collection in the left psoas. Percutaneous computed tomography-guided drainage was performed (B), with adequate positioning of a Sump (C), Ultrasound control showing important reduction in volume of collection (D)

Figure 2 shows a female, 22 years old, exchange student from Spain, with history of sub-acute back pain. Lumbar spine MR, T2-weighted, demonstrated elongated fluid collection in the left psoas, which was confirmed by US. Percutaneous CT-guided drainage was performed, with adequate positioning of a Sump drain, and aspiration of 130mL of thick bloody fluid. rt-PA was administrated inside the drain, in order to make the collection thinner. Control US after 4 days showed important reduction in collection volume. Patient was discharged with the drain and returned to her country. The final culture was positive for *S. aureus.*

**Figure 3:**
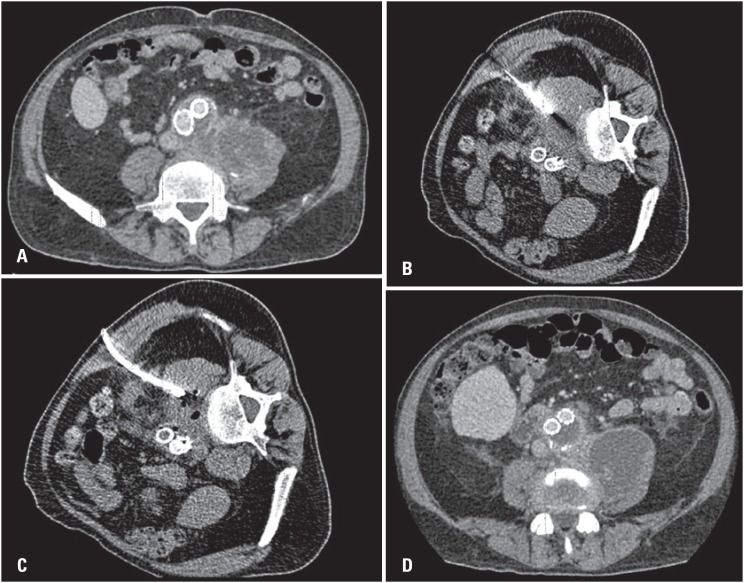
Contrast-enhanced abdominal computed tomography. (A) A fluid collection inside left psoas, surrounding aortoiliac graft, (B) Percutaneous computed tomography-guided drainage was performed using Seldinger technique, (C) Final imaging control demonstrating a well-positioned Dawson-Mueller drain, (D) Detection of a new fluid collection on the same site

Figure 3 demonstrates a male patient, 68 years old, with a history of endovascular aneurysm repair, admitted on emergency department with fever and abdominal pain. He was submitted to contrast-enhanced abdominal CT, which identified a fluid collection inside left psoas, surrounding aortoiliac graft. Computed tomography guided percutaneous drainage was made, using Seldinger technique, with aspiration of 90mL of purulent fluid. Patient was discharged in good clinical conditions after 8 days, but symptoms returned after 2 months. Another percutaneous drainage was performed after detection of a new fluid collection on the same site, which did not recur.

The success rate of percutaneous procedures was 71.5%, and two patients required reintervention due to recurrence of abscesses. Results are summarized in table 1.

**Table 1:** Summary of patients submitted to computed-tomography- and ultrasound-guided drainage of psoas abscess

Date of procedure	Sex	Age	Symptoms	Drain	Drain volume (mL)	Culture	Duration of drainage (days)
November 13, 2013	Male	76	Fever, abdominal pain, and back pain	Dawson-Mueller	35	*Pseudomonas aeruginosa*	9
June 27, 2014	Male	76	Abdominal pain	Dawson-Mueller	10	*Pseudomonas aeruginosa*	5
October 15, 2015	Male	68	Fever and abdominal pain	Dawson-Mueller	90	*Salmonella spp.*	8
January 1^st^, 2016	Male	68	Fever and abdominal pain	Sump	120	Negative	10
April 26, 2016	Female	22	Abdominal pain and back pain	Sump	130	*Staphylococcus aureus*	4
July 22^nd^, 2016	Male	92	Fever and abdominal pain	Dawson-Mueller	15	*Escherichia coli*	12
August 2^nd^, 2016	Male	54	Fever, abdominal pain and back pain	Dawson-Mueller	30	*Pseudomonas aeruginosa*	10

## DISCUSSION

Undrained IPA have high mortality rates, varying from 50 to 100%.^(^ ^6^ ^,^ ^7^ ^)^ Death is usually associated to sepsis. Needle aspiration alone is frequently unsuccessful and shows high recurrence rates.^(^ ^8^ ^)^

Historically, the treatment of IPA consisted of the surgical approach through retroperitoneal access, with removal of the abscess and necrosis, associated to adequate antibiotic therapy.^(^ ^9^ ^)^ Currently, with improvements in imaging techniques and greater expertise of radiologists on minimally invasive techniques, this approach has been preferred, due to lower morbidity and mortality and shorter hospital stay. Limitations concerning the method comprise patients with severe sepsis, who require a more immediate resolution of the abscess, and those presenting thick collections (although this limitation has been superseded with the use of fibrinolytic agents).^(^ ^10^ ^)^ Also, in patients with IPAs secondary to an underling abdominal condition (such as diverticulitis), surgical approach is preferable to address the underling cause.^(^ ^11^ ^)^

In our service, most cases are drained under CT guidance and using Seldinger technique, which consists of the utilization of a guidewire to acquire better positioning control and lower complication rates. However, it can be made using trocar technique, which consists of single-puncture drainage, and often saves time.^(^ ^12^ ^)^

There are no absolute contraindications for percutaneous drainages. Main relative contraindications are uncorrectable coagulopathy, lack of safe access (that can be solved using decubitus change and hydrodissection and/or pneumodissection) and lack of patient cooperation (sedation can be made by anesthesia team, if needed). The main advantages of this minimally-invasive technique are avoiding general anesthesia and surgical stress, thus reducing morbidity.^(^ ^13^ ^)^

Our series is small but should be considered representative, since this is a recent approach, not widespread yet, as compared to surgical treatments, despite its effectiveness, lower cost, less invasiveness and lower morbidity. In our series the mean catheter duration was 8.3 days, similar to rates reported in the literature (7 to 28 days), since longer duration can lead to fistula formation.^(^ ^14^ ^)^ Our service has no case of fistula. Periodic US or CT were made each 3 to 4 days, to assess drain position and eventual need of correcting it.

Complete cure of abscess with percutaneous drainage was achieved in five patients (71.5%), with a recurrence rate of 28.5%, which is corroborated by the literature, with rates from 14% to 29%.^(^ ^14^ ^)^ The clinical and radiological improvement, with no evidence of recurrence at 2 years or more, is considered a healed status.^(^ ^15^ ^)^

## CONCLUSION

Percutaneous image-guided drainage of iliopsoas abscess is a minimally invasive, efficient and safe procedure, with a good recovery and lower global costs; in many cases, it could avoid surgeries involving more morbidity and mortality risks. It is an important alternative for patients who cannot undergo surgery, due to poor clinical conditions or other contraindications.
